# Supplementary material for: The ISG Atlas: a loss-of-function analysis characterizes antiviral properties of interferon stimulated genes
Source: Nat Commun. 2026 May 8;17:4206. doi: 10.1038/s41467-026-72732-x (PMC13156316; doi:10.1038/s41467-026-72732-x)
Supplement: Supplementary file 1 — Supplementary Information [file 41467_2026_72732_MOESM1_ESM.pdf]

Supplementary information for:

The ISG Atlas: A Loss-Of-Function Analysis Characterizes Antiviral Properties of Interferon Stimulated Genes

Authors

Karsten Krey[1,§], Jennifer Risso-Ballester[1,§], Sabri Hamad[1], Susanne Maidl[1], Sara Bilekova[2,3,4], Quirin Emslander[1], Melissa Verin[1,5], Sarah Mundigl[1], Alexandrina Cernat[1], Antonio Piras[1], Valter Bergant[1], Vincent Grass[1], Andreas Pichlmair[1,5,6]

Affiliations

[1] Institute of Virology, School of Medicine and Health, Technical University of Munich, Munich, Germany

[2] Institute of Diabetes and Regeneration Research (IDR), Helmholtz Diabetes Center, Munich, Germany

[3] German Center for Diabetes Research (DZD), Neuherberg, Germany

[4] Technical University of Munich, School of Medicine, Munich, Germany

[5] Systems Virology, Institute of Virology, Helmholtz Center Munich, Munich, Germany

[6] German Center for Infection Research (DZIF), Munich Partner Site, Munich, Germany

*Correspondence: andreas.pichlmair@tum.de

[§] These authors contributed equally

Supplementary items: Supplementary Figures 1-8

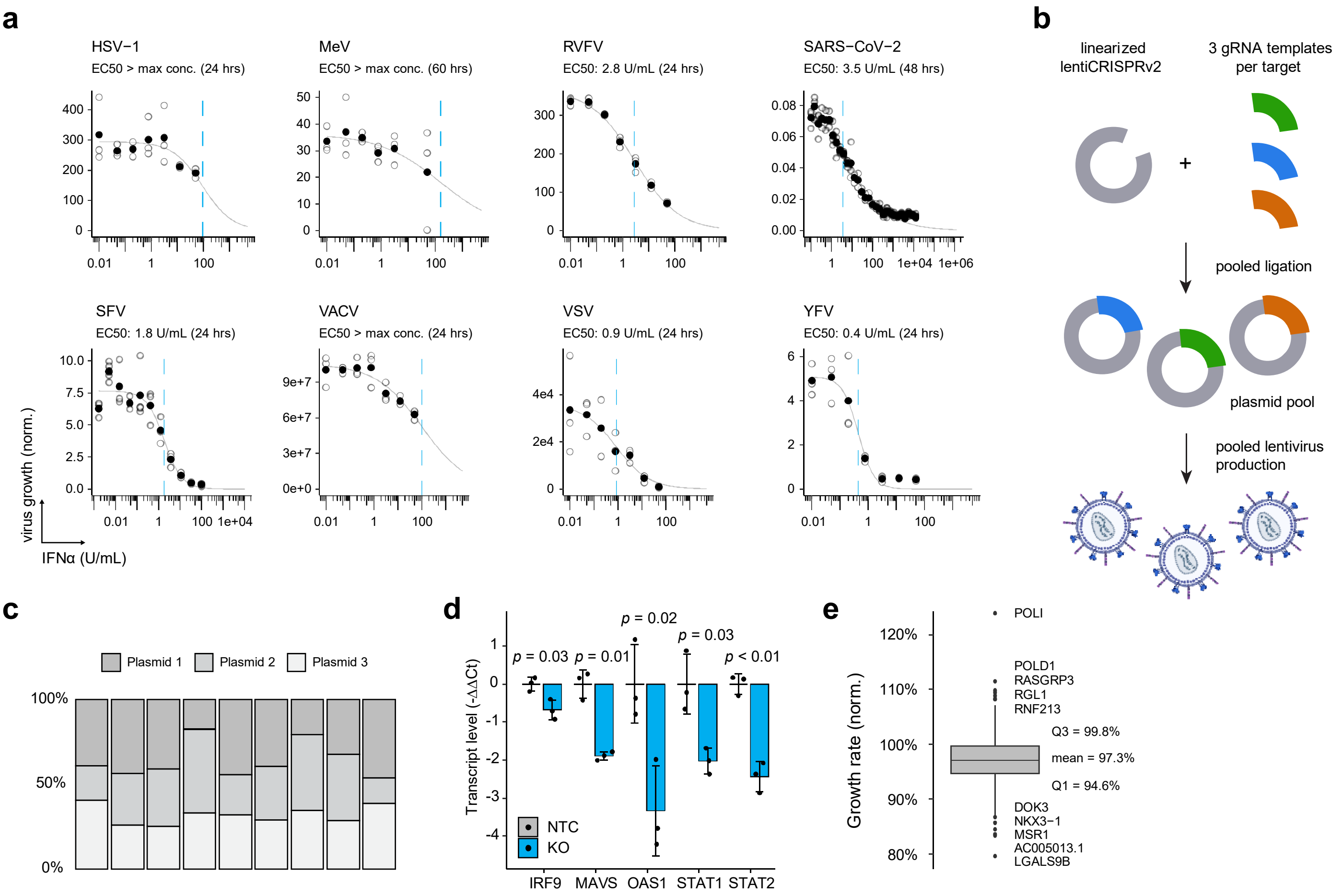

Supplementary Figure 1 | Generation of ISG Knockout Cells.

a) Dose-response curves of IFNα for the eight reporter viruses assessed via reporter fluorophore expression at the indicated time post infection. Black dots represent the mean of n = 3 biological replicates (empty dots). EC50 values are indicated by the cyan line. **b)** Schematic of the cloning strategy. For each ISG target, three guide sequences were pooled and ligated into a linearized lentiCRISPRv2 plasmid. Lentivirus particles were then produced and used to transduce A549 cells. Created in BioRender. Pichlmair, A. (2026) https://BioRender.com/1afh7fg. **c)** Relative distribution of guide sequence plasmids within randomly selected plasmid pools. The target region of the plasmid pools was quantified by qPCR using a U6-fwd and the reverse guide sequence as primers. **d)** Transcript levels of selected ISGs in KO and NTC A549 cells following IFNα treatment, measured by RT-qPCR. RPLP0-normalized values were normalized to the mean of the corresponding NTC group (−ΔΔCt). Mean ± SD (n = 3 biologically independent experiments). Statistical significance was assessed using a two-tailed, two-sample Welch's *t*-test. **e)** Growth rates of the 285 KO cell lines compared to the median growth rate of five NTC cell lines. The growth rates are derived from the accumulation of H2B-eGFP/mRFP signal of uninfected cells. KO cell lines for which growth rates were reduced by more than 25% compared to NTC cells were excluded from the analysis. (n = 4 biologically independent samples).

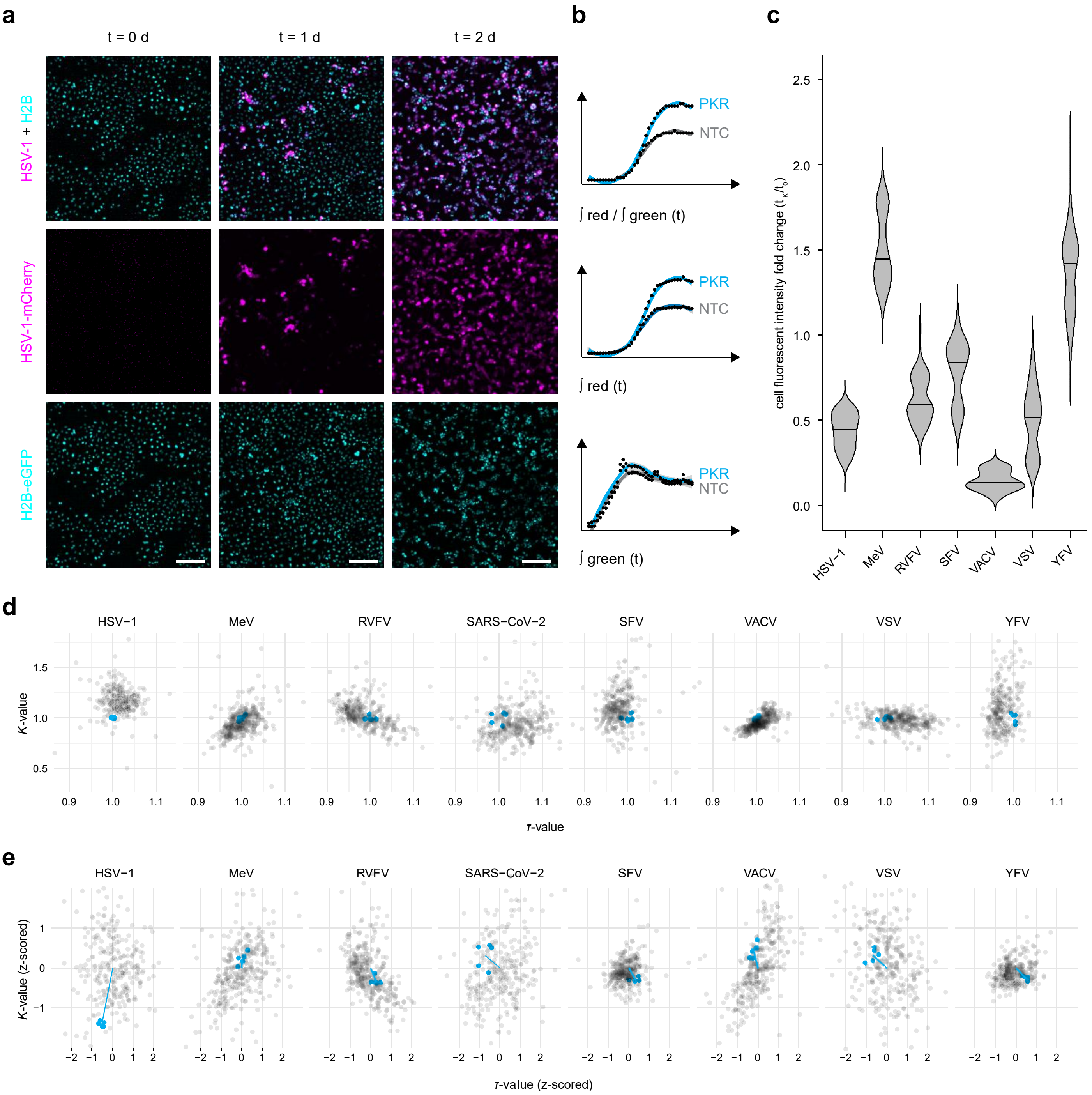

Supplementary Figure 2 | Strategy to Evaluate ISG Effects.

a) Live-cell fluorescence microscopy images of A549-NTC H2B-eGFP (cyan) infected with an HSV-1-mCherry reporter virus (magenta) for the indicated time. Scale bar: 200 µm. **b)** Time-course of the integrated intensities of the green and red fluorescence channel from HSV-1-mCherry infected A549-NTC and A549-EIF2AK2 (PKR) KO cells, with the ratio of red over green fluorescence indicating the progression of infection, following fitting to a logistic growth curve. **c)** Violin plots of H2B-RFP or H2B-GFP fluorescence intensity fold-change (t_k/t_0) in 100 randomly selected KO cell lines per virus. Fold-change compares maximal viral signal (t_k) to seeding (t_0). Horizontal lines denote medians; shape widths indicate data density (n = 3 biologically independent experiments). **d)** Scatter plots of NTC-normalized *K*- and *τ*-values for each virus. The five NTC samples are highlighted in cyan. **e)** Z-score normalized *K*- and *τ*-values across all viruses from g). The offset from the expected to the measured mean of the NTC values is indicated with a cyan line.

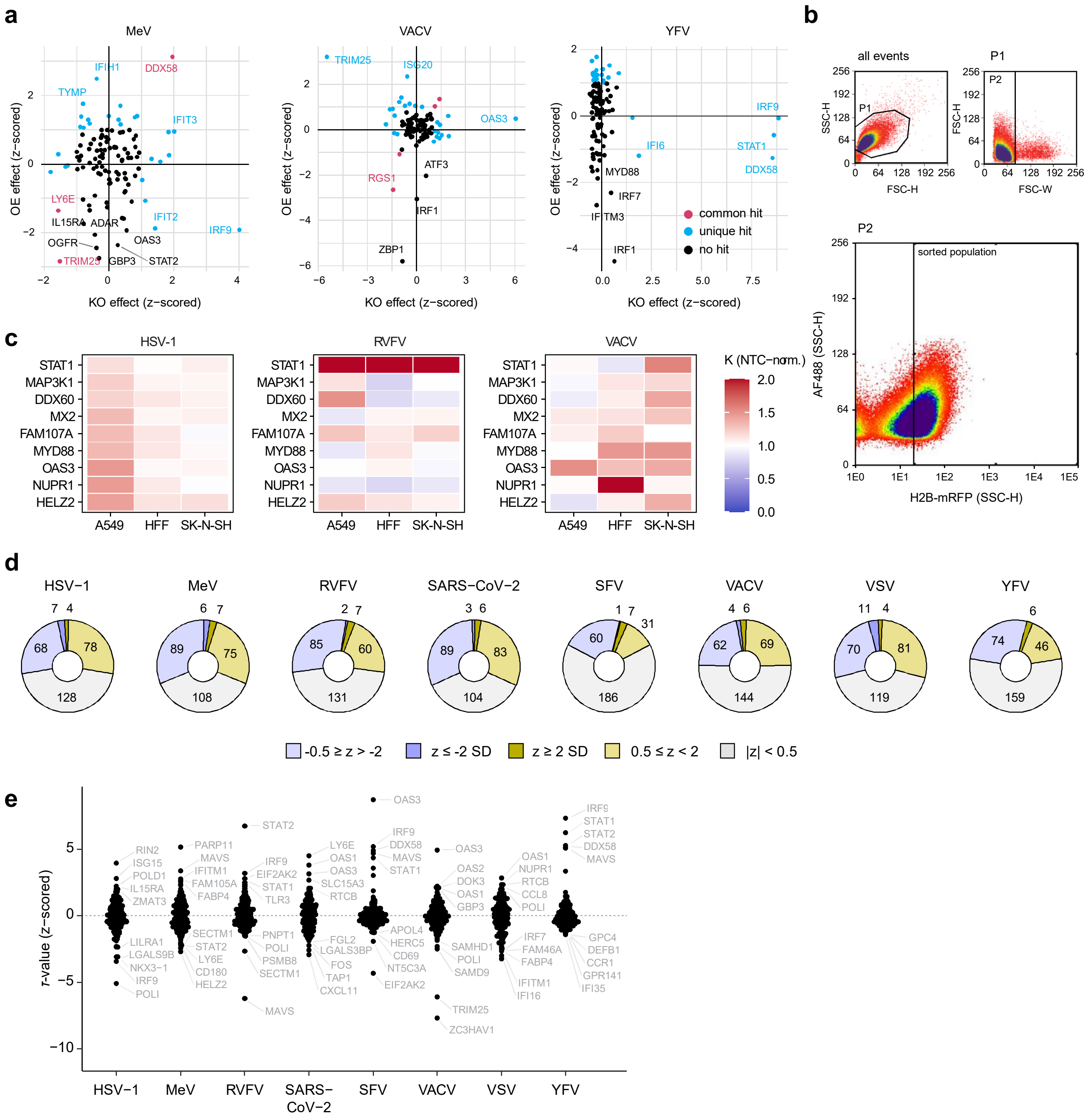

Supplementary Figure 3 | ISG Modulation on Viral Infection Dynamics.

a) Intersection of *K*-values from all KOs and previously published ISG overexpression screens. Both datasets were z-scored for each virus. ISGs with |z| ≥ 1 in both datasets were considered common hits, while ISGs with |z| ≥ 1 in only one dataset were considered unique. **b)** Flow cytometry gating strategy and sorting of H2B-expressing HFF cells. **c)** Analysis of the activity of a subset of ISGs in different cell models. CRISPR/Cas9 KOs in HFF-H2B-mRFP and SK-N-SH were generated, pre-treated with IFN and subsequently infected as described for A549. Infection was quantified from live-cell imaging using a normalized reporter readout: GFP/H2B-mRFP for HFF and GFP/phase-contrast (confluence) for SK-N-SH. For each replicate (n = 5 biologically independent experiments), the *K*-value was determined and normalized to NTC. The heat map shows the mean of the normalized effects alongside the A549 values. **d)** Categorical effects of ISG depletion based on their effect on τ-values: neutral (gray), minor effect on speed (0.5 < |z| ≤ 2 SDs, light blue and light yellow, respectively), and major effect on speed (|z| ≥ 2 SDs, blue and yellow, respectively). SD: standard deviation. **e)** Beeswarm plots of τ-values after z-scoring, showing all assessed KO effects.

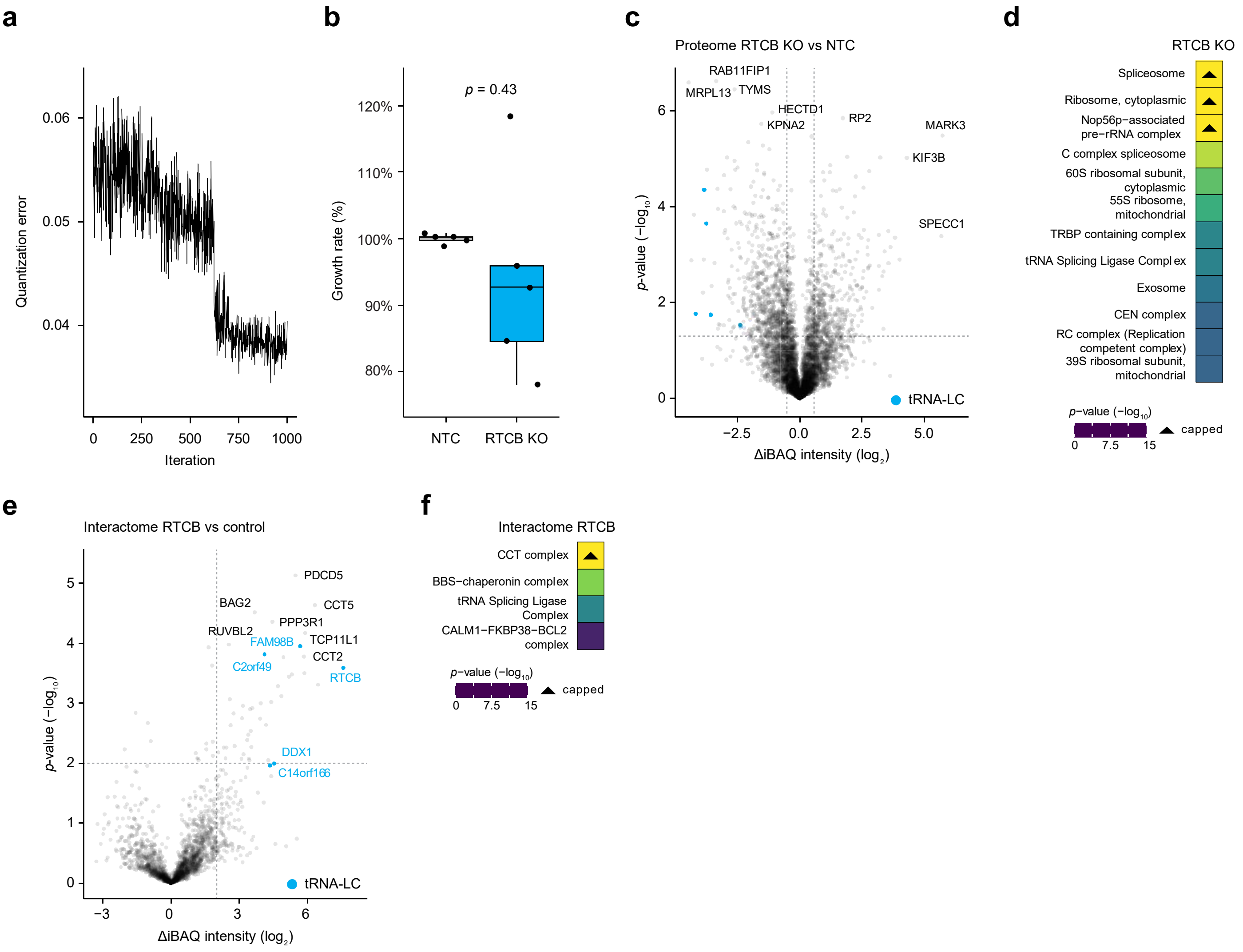

Supplementary Figure 4 | Self-Organizing Map and RTCB KO Characterization.

a) Quantization error trajectory of the self-organizing map model from Figure 3b, indicating the reduction of the learning rate and the convergence of the model to represent the input data in the multi-dimensional space. **b)** Growth rate data for RTCB KO cells compared to NTC cells within an observation time of 72 h, as shown in Figure 1c. The graph shows the mean of n = 5 biologically independent experiments for RTCB. For the NTC sample, the mean was calculated based on the five biological replicates for each of the five NTC samples. Box plots show the median (center line), first and third quartiles (box limits), and whiskers extending up to 1.5× the interquartile range. Two-sided, two-sample Welch's *t*-test. *p*-values ≤ 0.05 were considered significant. **c)** Volcano plot showing the changes of the cellular proteome in RTCB KO cells measured by mass spectrometry. Members of the tRNA ligase complex are highlighted in cyan. Two-tailed, two-sample Welch's *t*-test (n = 4 biologically independent samples). Protein groups with a $|\log_2 FC| \geq 0.5$ and a *p*-value ≤ 0.05 were considered significant. **d)** Gene set enrichment analysis based on the data in c) using g:Profiler and a one-sided hypergeometric test. *p*-values were adjusted for multiple comparisons using the g:SCS algorithm. The most significant CORUM terms are shown. *p*-values exceeding the maximum range are capped and indicated with a triangle. **e)** Volcano plot showing protein interactions identified by affinity-purification mass spectrometry (AP-MS) in A549 cells overexpressing RTCB. Subunits of the tRNA ligase complex are indicated in cyan. Data were analyzed using a two-tailed, two-sample Welch's *t*-test (n = 3 biologically independent samples). Protein groups with a $|\log_2 FC| \geq 2$ and a *p*-value ≤ 0.01 were considered significant and are indicated by a dotted line. **f)** Gene set enrichment analysis based on the data in e) using g:Profiler and a one-sided hypergeometric test. p-values were adjusted for multiple comparisons using the g:SCS algorithm. The most significant CORUM terms are shown. *p*-values exceeding the maximum range are capped and indicated with a triangle.

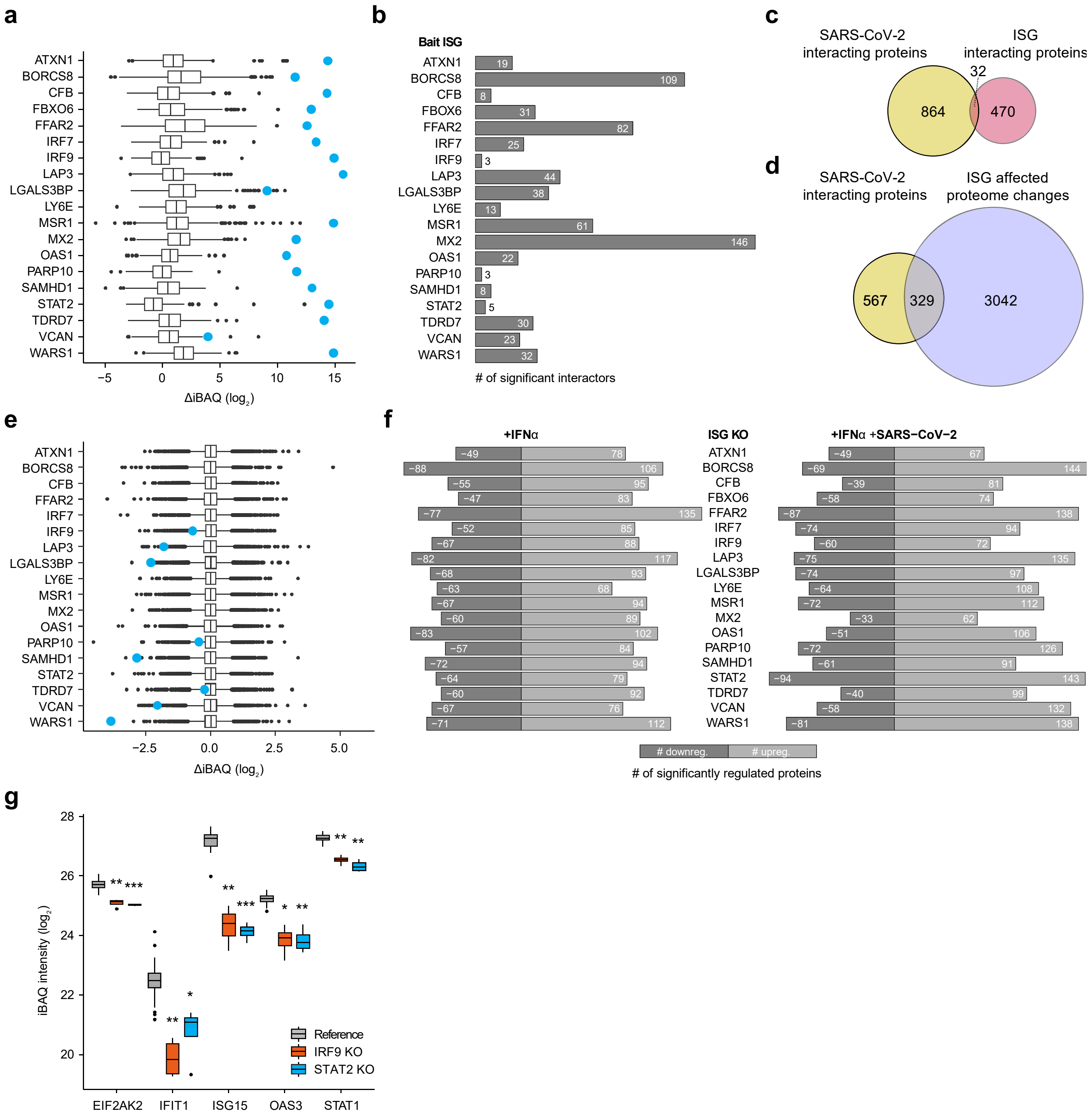

Supplementary Figure 5 | Proteomics Analysis of ISG Effects on the Cellular Proteome.

a) The indicated ISGs were expressed as V5-tagged fusion proteins in A549 cells, and V5 precipitates were subjected to liquid chromatography coupled with tandem mass spectrometry (LC-MS/MS) analysis. The graph shows the iBAQ fold change of the precipitated ISG in relation to control samples and enrichment of co-precipitated proteins. Protein abundance of overexpressed ISGs is shown in cyan. (n = 4 biologically independent samples). **b)** Number of proteins that were significantly enriched in the indicated ISG precipitates. Significant interactors were defined as log2FC ≥ 2 and $p \leq 0.01$. **c)** Intersection of proteins interacting with SARS-CoV-2 and with 19 ISGs from b). **d)** Intersection of SARS-CoV-2 interacting proteins with proteins regulated by expression of the 19 ISGs. **e)** ISG knockout (KO) cells were treated with IFNα (6.25 U/mL for 30 h) or pre-treated with IFNα (6 h with 6.25 U/mL) and subsequently infected with SARS-CoV-2 (MOI 3) for 24 h. Cellular protein expression profiles were analyzed by liquid chromatography coupled with tandem mass spectrometry (LC-MS/MS). The graph shows the iBAQ fold change of all quantified proteins in relation to global reference for IFN condition. The expression of the targeted ISG is marked in cyan. (n = 4 biologically independent experiments). **f)** Number of proteins that were significantly up- (light gray) or downregulated (dark gray) in the indicated ISG KO cells. Significant expression changes were defined as |log2FC| ≥ 2 and $p \leq 0.05$. **g)** Protein abundance (iBAQ values) of the indicated ISG for the KO of STAT2 and IRF9, compared to the global reference consisting of all other samples of the

same treatment group excluding the sample itself. Box plots show the median (center line), first and third quartiles (box limits), and whiskers extending up to 1.5× the interquartile range. A two-tailed, two-sample Welch's t-test was performed (n = 4 biologically independent experiments), followed by a false discovery rate (FDR) correction of the p-values to account for multiple comparisons. *: p-value < 0.05, **: p-value < 0.01, ***: p-value < 0.001.

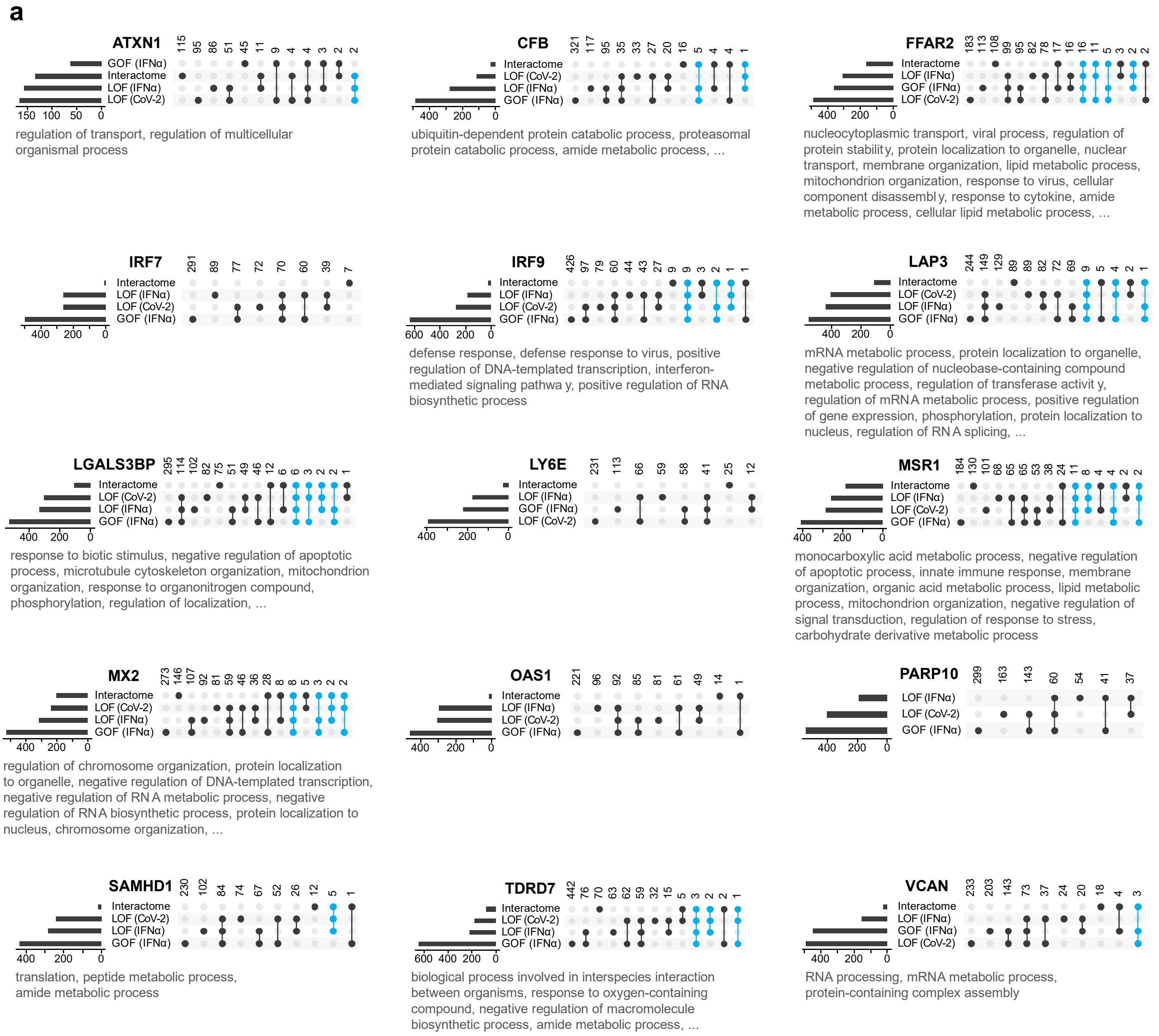

Supplementary Figure 6 | Intersection of Enrichment Terms of Proteomics Datasets.

a) UpSet plots for the indicated ISG showing the intersection of enriched GO:BP terms derived from the significantly enriched proteins of the datasets depicted in Figure 4d). The bar plots on the left of each UpSet plot show the total number of enriched GO:BP terms per dataset. The number of shared terms among datasets is shown at the top of each intersection. The terms present in at least two proteome datasets and the interactome dataset (cyan intersects) were selected and are shown below each plot. The terms were manually filtered for redundancy and generic terms.

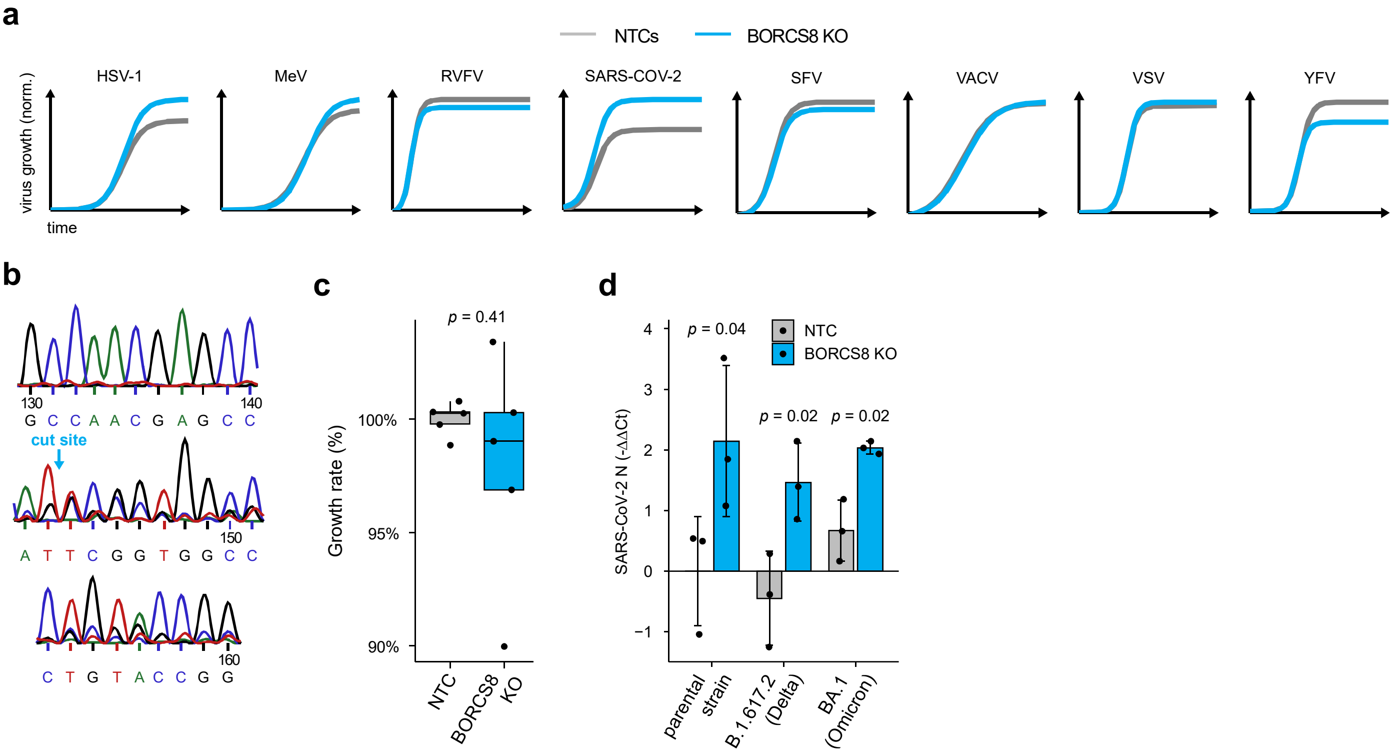

Supplementary Figure 7 | Virus Growth in BORCS8 KO Cells.

a) Virus-derived fluorophore signals measured by live-cell imaging normalized to the H2B-mRFP or -eGFP signal. The sigmoidal curves are based on the mean parameters of all replicates and depict the comparison between BORCS8 knockout (KO) (cyan) and the mean of five non-targeting control (NTC) samples (gray) for each virus. **b)** Sanger sequencing of the *BORCS8* gene locus, displaying overlapping peaks in the target region of CRISPR/Cas9 (cyan arrow). **c)** Growth rate of BORCS8 KO cells compared to NTC cells within an observation time of 72 h, as shown in Figure 1c. The graph shows the mean of n = 5 biological replicates for BORCS8. For the NTC sample, the mean was calculated based on the five biological replicates for each of the five NTC samples. Box plots show the median (center line), first and third quartiles (box limits), and whiskers extending up to 1.5× the interquartile range. Two-sided, two-sample Welch's *t*-test. **d)** SARS-CoV-2 mRNA expression of N measured by RT-qPCR, showing RPLP0-normalized values, further normalized to the mean of the NTC samples of the parental strain of SARS-CoV-2. Shown are the data for selected SARS-CoV-2 variants of concern (VoC). Two-tailed, two-sample Welch's *t*-test (n = 3 biologically independent experiments).

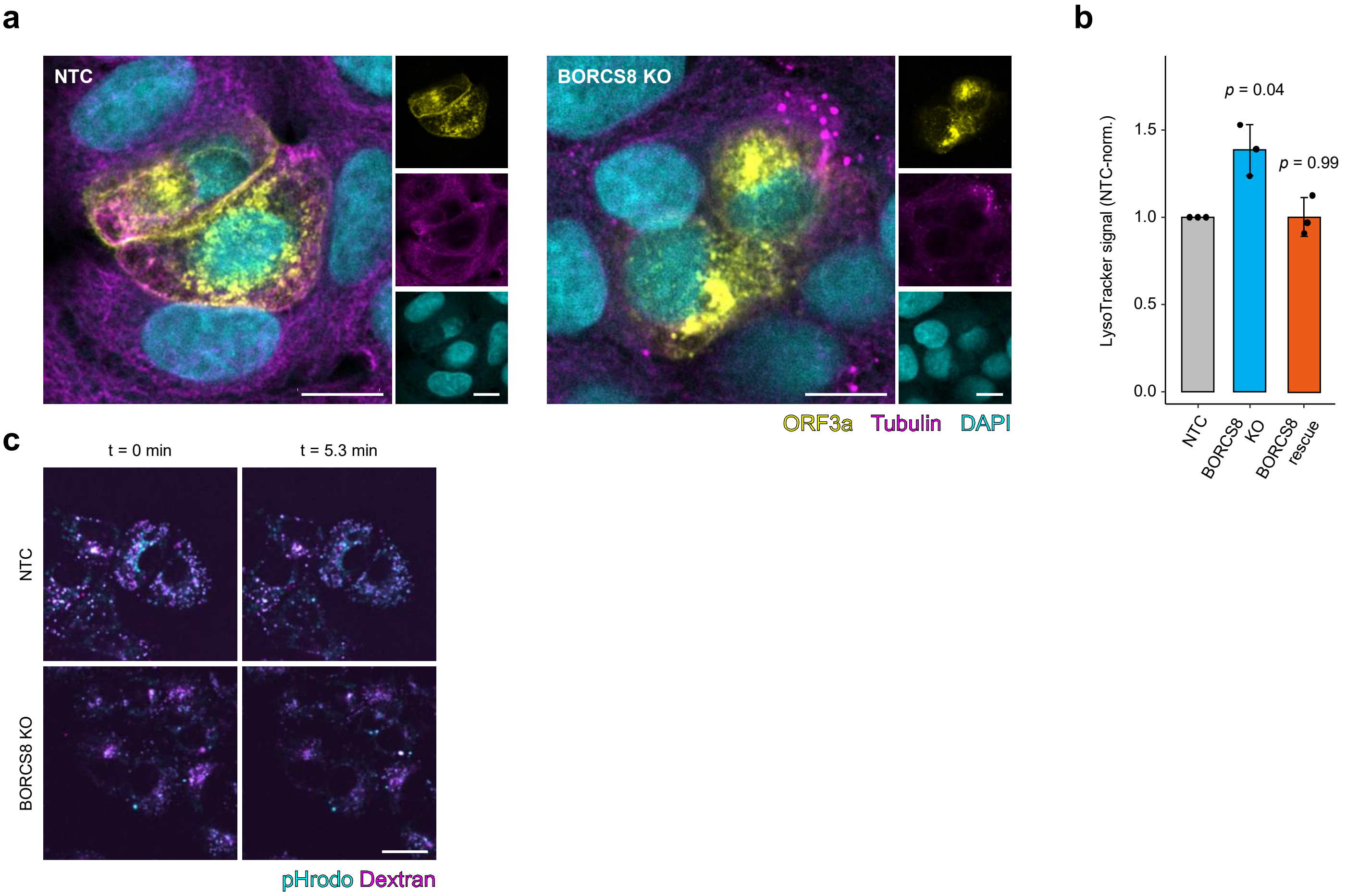

Supplementary Figure 8 | Impact of BORCS8 on Vesicular Transport and Lysosomal Exocytosis.

a) Representative Airyscan images of NTC and BORCS8 KO cells infected with SARS-CoV-2 and stained for ORF3a (SARS-CoV-2), α-Tubulin, and DAPI. Maximum intensity projection. Scale bar: 10 µm. **b)** Quantification of LysoTracker Red DND-99 signal in BORCS8 KO and BORCS8 rescue A549-ACE2 cells relative to NTC at 12 h post-staining. Data are presented as mean ± SD (n = 3 biologically independent experiments). Statistical significance was assessed using a one-sided, one-sample Welch's t-test. **c)** Representative images of the time-resolved colocalization of endocytosed cargo from Figure 7h) in NTC and BORCS8 KO cells stained for pHrodo Green and Dextran-AF647. *p*-values ≤ 0.05 were considered significant.
