# Supplementary material for: The ISG Atlas: a loss-of-function analysis characterizes antiviral properties of interferon stimulated genes
Source: Nat Commun. 2026 May 8;17:4206. doi: 10.1038/s41467-026-72732-x (PMC13156316; doi:10.1038/s41467-026-72732-x)
Supplement: Supplementary file 2 — Description of Additional Supplementary Files [file 41467_2026_72732_MOESM2_ESM.pdf]

Description of Additional Supplementary Files

Supplementary Data 1 | CRISPR/Cas9 Guide Sequences, Overexpression ORFs, and qPCR Primers.
Supplementary Data 2 | Infection Kinetics and Growth Rates for All ISGs and Viruses.
Supplementary Data 3 | Proteomics Data of RTCB Knockout Cells.
Supplementary Data 4 | Proteomics Data of RTCB Overexpressing Cells.
Supplementary Data 5 | Proteomics Data of Selected ISG KO Cells.
Supplementary Data 6 | Proteomics Data of Selected ISG Overexpressing Cells.
Supplementary Data 7 | Intersection of GO Terms From Different Proteomics Datasets.
Supplementary Data 8 | Interaction Score Calculations for Double Knockouts.
