# Supplementary material for: The ISG Atlas: a loss-of-function analysis characterizes antiviral properties of interferon stimulated genes
Source: Nat Commun. 2026 May 8;17:4206. doi: 10.1038/s41467-026-72732-x (PMC13156316; doi:10.1038/s41467-026-72732-x)
Supplement: Supplementary file 11 — Reporting Summary [file 41467_2026_72732_MOESM11_ESM.pdf]

nature portfolio

Corresponding author(s): Prof. Dr. Andreas Pichlmair

Last updated by author(s): 04/10/2026

Reporting Summary

Nature Portfolio wishes to improve the reproducibility of the work that we publish. This form provides structure for consistency and transparency in reporting. For further information on Nature Portfolio policies, see our Editorial Policies and the Editorial Policy Checklist.

Statistics

For all statistical analyses, confirm that the following items are present in the figure legend, table legend, main text, or Methods section.

n/a	Confirmed	
☐	☒	The exact sample size (*n*) for each experimental group/condition, given as a discrete number and unit of measurement
☐	☒	A statement on whether measurements were taken from distinct samples or whether the same sample was measured repeatedly
☐	☒	The statistical test(s) used AND whether they are one- or two-sided<br>*Only common tests should be described solely by name; describe more complex techniques in the Methods section.*
☐	☒	A description of all covariates tested
☐	☒	A description of any assumptions or corrections, such as tests of normality and adjustment for multiple comparisons
☐	☒	A full description of the statistical parameters including central tendency (e.g. means) or other basic estimates (e.g. regression coefficient) AND variation (e.g. standard deviation) or associated estimates of uncertainty (e.g. confidence intervals)
☐	☒	For null hypothesis testing, the test statistic (e.g. *F*, *t*, *r*) with confidence intervals, effect sizes, degrees of freedom and *P* value noted<br>*Give P values as exact values whenever suitable.*
☒	☐	For Bayesian analysis, information on the choice of priors and Markov chain Monte Carlo settings
☒	☐	For hierarchical and complex designs, identification of the appropriate level for tests and full reporting of outcomes
☒	☐	Estimates of effect sizes (e.g. Cohen's *d*, Pearson's *r*), indicating how they were calculated

Our web collection on statistics for biologists contains articles on many of the points above.

Software and code

Policy information about availability of computer code

Data collection	IncuCyte S3 software (2020C Rev1)
Data analysis	If not otherwise specified, the data was analyzed using R (version 4.2.2, 2022-10-31 ucrt) and R studio (version 2023.06.1, Build 524). The following R packages and versions were used: broom (1.0.5), circlize (0.4.15), ComplexHeatmap (2.14.0), EBImage (4.40.1), ggpubr (0.6.0), ggrepel (0.9.3), gprofiler2 (0.2.2), ggsignif (0.6.4), glmnet (4.1-8), GMSimpute (0.0.1.0), heatmaply (1.4.2), kohonen (3.0.12), pheatmap (1.0.12), rstatix (0.7.2), splines (4.2.2), tidyverse (2.0.0). The scripts for curve fitting of IncuCyte image data is available at https://github.com/innatelab/isg-atlas.

For manuscripts utilizing custom algorithms or software that are central to the research but not yet described in published literature, software must be made available to editors and reviewers. We strongly encourage code deposition in a community repository (e.g. GitHub). See the Nature Portfolio guidelines for submitting code & software for further information.

Data

Policy information about availability of data

All manuscripts must include a data availability statement. This statement should provide the following information, where applicable:

- Accession codes, unique identifiers, or web links for publicly available datasets
- A description of any restrictions on data availability
- For clinical datasets or third party data, please ensure that the statement adheres to our policy

The mass spectrometry proteomics data have been deposited to the ProteomeXchange Consortium via the PRIDE partner repository. This includes the following

datasets: proteome of RTCB KO cells (PXD045812), affinity purification of cells overexpressing RTCB (PXD045929); proteomes of ISG KOs (PXD045783) and affinity purification of overexpressed ISGs (PXD045812).

Research involving human participants, their data, or biological material

Policy information about studies with human participants or human data. See also policy information about sex, gender (identity/presentation), and sexual orientation and race, ethnicity and racism.

Reporting on sex and gender	n/a
Reporting on race, ethnicity, or other socially relevant groupings	n/a
Population characteristics	n/a
Recruitment	n/a
Ethics oversight	n/a

Note that full information on the approval of the study protocol must also be provided in the manuscript.

Field-specific reporting

Please select the one below that is the best fit for your research. If you are not sure, read the appropriate sections before making your selection.

[x] Life sciences [ ] Behavioural & social sciences [ ] Ecological, evolutionary & environmental sciences

For a reference copy of the document with all sections, see nature.com/documents/nr-reporting-summary-flat.pdf

Life sciences study design

All studies must disclose on these points even when the disclosure is negative.

Sample size	Sample sizes were chosen based on prior experience
Data exclusions	No data was excluded
Replication	All described data was replicated independently at least two more times
Randomization	No randomization of data was performed, as it was not applicable to this study
Blinding	No blinding was performed, as we considered it to be unnecesary for this study

Reporting for specific materials, systems and methods

We require information from authors about some types of materials, experimental systems and methods used in many studies. Here, indicate whether each material, system or method listed is relevant to your study. If you are not sure if a list item applies to your research, read the appropriate section before selecting a response.

Materials & experimental systems

n/a	Involved in the study	
[ ]	[x]	Antibodies
[ ]	[x]	Eukaryotic cell lines
[x]	[ ]	Palaeontology and archaeology
[x]	[ ]	Animals and other organisms
[x]	[ ]	Clinical data
[x]	[ ]	Dual use research of concern
[x]	[ ]	Plants

Methods

n/a	Involved in the study	
[x]	[ ]	ChIP-seq
[ ]	[x]	Flow cytometry
[x]	[ ]	MRI-based neuroimaging

Antibodies

Antibodies used	Primary antibodies: α-LAMP1 (Cell Signaling Technology, 9091S, 1:400), α-tubulin (Sigma Aldrich, MAB186, 1:400), α-ORF3a (Abcam, ab280953, 1:500), α-NP (Sino Biological, 40143-MM05, 1:500). Secondary antibodies: donkey anti-rabbit Alexa Fluor 488 (Invitrogen, A21206, 1:600), donkey anti-rat Cy3, (Jackson ImmunoResearch, 712-165-153, 1:600)

Validation	All antibodies were validated by the manufacturer

Eukaryotic cell lines

Policy information about cell lines and Sex and Gender in Research

Cell line source(s)	A549 (ATCC, CCL-185), A549-ACE274, HEK293T (ATCC, CRL-11268) , Vero E6 (ATCC, CRL-1586), HFF (ATCC, SCRC-1041), SK-N-SH (ATCC, HTB-11) and Calu-3 cells (ATCC, HTB-55) (kindly provided by Stephan Pöhlmann, Deutsches Primatenzentrum, Münster, Germany, and Stephan Ludwig, University of Münster, Germany)
Authentication	Authentication was performed by STR profiling
Mycoplasma contamination	All cell lines were regularly tested to be mycoplasma-free
Commonly misidentified lines (See ICLAC register)	not applicable

Plants

Seed stocks	not applicable
Novel plant genotypes	not applicable
Authentication	not applicable

Flow Cytometry

Plots

Confirm that:

- [x] The axis labels state the marker and fluorochrome used (e.g. CD4-FITC).
- [x] The axis scales are clearly visible. Include numbers along axes only for bottom left plot of group (a 'group' is an analysis of identical markers).
- [x] All plots are contour plots with outliers or pseudocolor plots.
- [x] A numerical value for number of cells or percentage (with statistics) is provided.

Methodology

Sample preparation	no preparation/staining was required
Instrument	Beckman Coulter Astrios EQ
Software	Summit v6
Cell population abundance	Cells expressing the highest level of the expressed fluorescent H2B-fusion protein were sorted. This was confirmed by fluorescence microscopy
Gating strategy	Cells corresponding to the live cell population were gated for singlets and further for high fluorophor expression

- [x] Tick this box to confirm that a figure exemplifying the gating strategy is provided in the Supplementary Information.
